# Return to work of workers without a permanent employment contract, sick-listed due to a common mental disorder: design of a randomised controlled trial

**DOI:** 10.1186/1471-2458-14-594

**Published:** 2014-06-12

**Authors:** Lieke Lammerts, Sylvia J Vermeulen, Frederieke G Schaafsma, Willem van Mechelen, Johannes R Anema

**Affiliations:** 1 Department of Public and Occupational Health, EMGO + Institute for Health and Care Research, VU University Medical Centre, P.O. Box 7057, Amsterdam 1007 MB, The Netherlands; 2 Research Centre for Insurance Medicine AMC-UMCG-UWV-VUmc, Amsterdam, The Netherlands

**Keywords:** Return to work, Intervention, Sickness absence, Common mental disorders, Unemployed workers, Temporary agency workers, Fixed-term contract workers, Vulnerable workers, Occupational health care, Social security, Randomised controlled trial

## Abstract

**Background:**

Workers without a permanent employment contract represent a vulnerable group within the working population. Mental disorders are a major cause of sickness absence within this group. Common mental disorders are stress-related, depressive and anxiety disorders. To date, little attention has been paid to effective return to work interventions for this type of sick-listed workers. Therefore, a participatory supportive return to work program has been developed. It combines elements of a participatory return to work program, integrated care and direct placement in a competitive job.

The objective of this paper is to describe the design of a randomised controlled trial to evaluate the cost-effectiveness of this program compared to care as usual.

**Methods/Design:**

The cost-effectiveness of the participatory supportive return to work program will be examined in a randomised controlled trial with a follow-up of twelve months.

The program strongly involves the sick-listed worker in the identification of obstacles for return to work and possible solutions, resulting in a consensus based action plan. This plan will be used as a starting point for the search of suitable competitive employment with support of a rehabilitation agency. During this process the insurance physician of the sick-listed worker contacts other caregivers to promote integrated care.

Workers eligible to participate in this study have no permanent employment contract, have applied for a sickness benefit at the Dutch Social Security Agency and are sick-listed between two and fourteen weeks due to mental health problems.

The primary outcome measure is the duration until first sustainable return to work in a competitive job. Outcomes are measured at baseline and after three, six, nine and twelve months.

**Discussion:**

If the participatory supportive return to work program proves to be cost-effective, the social security system, the sick-listed worker and society as a whole will benefit. A cost-effective return to work program will lead to a reduction of costs related to sickness absence. For the sick-listed worker a cost-effective program results in earlier sustainable return to work, which can be associated with both social and health benefits.

**Trial registration:**

The trial registration number and date is NTR3563, August 7, 2012.

## Background

### The need for a return to work perspective

Workers without a permanent employment contract, such as unemployed workers, temporary agency workers and fixed-term contract workers, represent a vulnerable group within the working population. Unemployment seems to be associated with poor health [1,2] and research suggests that flexible work arrangements might share some of these negative consequences for workers’ health with unemployment [3]. To illustrate, in their systematic review on temporary employment and health Virtanen et al. [4] found evidence for an association between temporary employment and increased psychological morbidity.

In most European countries the non-permanent employment rate has increased during the last two decades [5]. In the Netherlands in 2012 almost a quarter of the active labour force was working on a temporary basis, compared to almost 18 percent in 2001 [6]. A major reason for the increase in flexible employment relationships is the need for companies to adjust easily to international developments [5]. Also, due to the shrinking Dutch economy in the last couple of years, more people have become unemployed [7].

In the Netherlands, the Dutch Social Security Agency (SSA) is responsible for occupational health care (OHC) of sick-listed workers who have no (longer an) employment contract. The SSA carries out the Sickness Benefit Act, which provides supportive income, i.e. sickness benefit, for these types of sick-listed workers [8].

In their report of 2011 on characteristics of prolonged sick-listed workers without a permanent employment contract, the Dutch SSA mentioned mental disorders as the most frequently diagnosed disorders among this group [9]. Within the European region mental health problems are increasingly acknowledged as a major public health concern [10,11]. They affect at least one in four people in the European region at some point in their lives [11]. Moreover, a recent study on the mental health consequences of the economic recession in European countries suggests that the impact of loss of employment on people with mental health problems is more severe than on people without mental health problems [12]. In the Netherlands, common mental disorders (CMDs) are stress-related disorders, depressive disorders and anxiety disorders [13,14].

Compared to sick-listed workers with a permanent employment contract, in the Netherlands sick-listed workers without a permanent employment contract perceive their health status more negatively and encounter more psychosocial barriers for their return to work (RTW) [15,16]. Moreover, sick-listed workers without a permanent employment contract experience a greater distance to the labour market compared to sick-listed employees, because there is often no workplace to return to [15]. To date, only little attention has been paid to the development of RTW interventions for sick-listed workers without a permanent employment contract who experience work limitations due to a CMD [17]. The aim of this study was to develop a RTW intervention for this group of sick-listed workers and to investigate the cost-effectiveness of this intervention.

### The development of a participatory supportive return to work intervention

The development of a RTW intervention for workers without a permanent employment contract who are sick-listed due to a CMD was based on an already existing participatory RTW program. Key elements of this intervention are active participation and strong commitment of both the sick-listed worker and his supervisor in a stepwise process to identify and solve obstacles for RTW, resulting in a consensus based RTW action plan [18]. We examined the strengths, weaknesses and points for improvement of the participatory RTW program reported in the literature. In addition, important stakeholders were consulted to assess the need for a participatory RTW program for workers without a permanent employment contract, sick-listed due to a CMD. Interviews were held with managers and professionals of the Dutch SSA, representatives of three Dutch rehabilitation agencies and representatives of the Dutch mental health care sector. To investigate the needs of the intended target group of the RTW program, results from a survey among 810 sick-listed workers without a permanent employment contract who applied for a sickness benefit at the Dutch SSA were used [16].

Studies on the effectiveness of the participatory RTW program reveal that this program significantly reduced time to RTW of employees two to six weeks sick-listed due to low back pain and of employees two to eight weeks sick-listed due to distress who at baseline intended to return to work despite symptoms, compared to care as usual [19,20]. Vermeulen and colleagues were the first who studied the cost-effectiveness of this program for sick-listed workers without a permanent employment contract, namely for temporary agency workers and unemployed workers sick-listed between two and eight weeks due to a musculoskeletal disorder [21]. Because these sick-listed workers had no (longer a) workplace to return to, placement in a matching temporary (therapeutic) workplace with ongoing supportive benefit by the SSA was added to the original participatory RTW program. The median duration until sustainable first RTW was 161 days for temporary agency workers and unemployed workers who had received the intervention and 299 days in the usual care group [22].

The results of the study of Vermeulen et al. indicate that the participatory RTW program is also an effective RTW intervention for sick-listed workers without a permanent employment contract. However, in this study the SSA paid supportive benefit (from public money) during placement in a temporary (therapeutic) workplace. This made the intervention more costly than usual care from the social insurer’s perspective [23]. Therefore, in the present RTW program for sick-listed workers without a permanent employment contract who are sick-listed due to a CMD, the focus has been shifted from placement in a temporary (therapeutic) workplace with ongoing supportive benefit to direct placement in a competitive job. Direct placement in a competitive job has already shown to improve the RTW of people with severe mental illness as part of Individual placement and Support (IPS) programs [24,25]. The essence of IPS is to first place in suitable competitive employment and then train by offering personal guidance at the workplace [24,26]. Moreover, results of the survey of Van der Burg et al. show that placement in a suitable job during sickness absence positively affected sustainable RTW of sick-listed workers without an employment contract who applied for a sickness benefit [16].

Another practice that has been incorporated in the present participatory RTW program is an integrated care approach. The participatory supportive RTW program has been developed in line with a Dutch covenant between the SSA and the mental health care sector that was signed recently. This covenant has the mutual aim to improve the (occupational) participation of sick-listed workers with mental disorders. The importance of integration of mental and occupational health care has also been emphasized in several studies. To illustrate, Olesen and colleagues [27] suggest in their study about mental health and employment that policies to promote and maintain workforce participation should be incorporated in mental health care, to prevent social exclusion of the sick-listed worker and to achieve a more sustainable contribution of this vulnerable group of workers to the labour force. According to a study of Anema and colleagues, in the Netherlands, communication between occupational health and other health care professionals, such as mental health care professionals, has been limited [28]. These findings were confirmed by the insurance physicians we interviewed. They acknowledged the importance of collaboration with the caregivers of their clients, but experienced obstacles in approaching these caregivers. In the present participatory RTW program, the insurance physicians are asked to actively involve the caregiver(s) of the sick-listed worker in their advice on RTW possibilities. Communication formats, e.g. a letter with a contact request and information about the study, are provided to the insurance physicians to facilitate making contact with the caregiver(s) of the sick-listed worker.

Hence, direct placement in a competitive job and an integrated care approach were integrated into the initial participatory RTW program, resulting in a participatory supportive RTW program aimed for workers without a permanent employment contract who are sick-listed due to a CMD.

### Objective

The objective of this paper is to describe the design of a randomised controlled trial (RCT). This study aims to investigate the cost-effectiveness of the participatory supportive RTW program for workers without a permanent employment contract who are sick-listed due to a CMD on the duration until first sustainable RTW in a competitive job, compared to usual OHC.

## Methods/Design

The design of the RCT will be described following the guidelines for reporting randomised trials provided by the CONSORT statement [29].

### Trial design

The study design consists of a RCT with two arms: a control group and an intervention group. Both the control group and the intervention group will receive usual OHC. In addition, the intervention group will be guided according to the new participatory supportive RTW program. Measurements will take place at baseline and after three, six, nine and twelve months.

Seven front offices of the Dutch SSA, ‘The Dutch Institute for Employee Benefit Schemes’ (in Dutch: ‘Uitvoeringsinstituut Werknemersverzekeringen’), will participate in the RCT together with three vocational rehabilitation agencies operating on national level. Each participating SSA office will be asked to assign two intervention teams of OHC professionals to participate in the study. These intervention teams will be trained to guide intervention group respondents according to the participatory supportive RTW program.

Randomisation will take place at the level of the participant. A separate block randomisation table will be generated for each SSA district. Beforehand, the SSA front offices will be divided into three regional districts.

The trial design, procedures and informed consent have been approved by the Medical Ethics Committee of the VU University Medical Centre (Amsterdam, The Netherlands).

Participation in de study will be voluntary and will only be possible when the participant signs informed consent.

A project team will be formed to monitor the conduct of the trial. This project team will consist of the researchers, representatives of the SSA and representatives of the vocational rehabilitation agencies. Towards the stakeholders and participants, the RCT is entitled the ‘Co-WORK’ (in Dutch: ‘SamenWERK’) study.

The trial has been registered at the Dutch Trial Register (‘Nederlands Trial Register’) on August 7, 2012.

### Study population

Workers eligible to participate in the study are sick-listed workers without a permanent employment contract who have applied for a sickness benefit at the Dutch SSA, e.g. sick-listed unemployed workers, temporary agency workers and workers with an expired fixed-term employment contract, in the working age range (18–64 years), and sick-listed between two and fourteen weeks with mental health problems as main reason for their sickness benefit claim.

Earlier research on the effectiveness of a participatory approach suggested that sick-listed workers who believe they should be fully recovered before they RTW, require another RTW intervention [20,22]. Therefore, not having the intention to RTW in case health complaints are still experienced is an exclusion criterion for participating in this study. Other exclusion criteria are: 1. not being able to complete questionnaires written in the Dutch language; 2. having a conflict with the SSA regarding a sickness benefit claim or a long-term disability claim; 3. the presence of a legal conflict, e.g. an ongoing injury compensation claim; 4. a sickness absence episode due to a CMD within one month before the current sickness benefit claim; 5. already having received usual OHC since the start of the current sickness absence period, 6. Pregnancy, up until three months after delivery and 7. no signed informed consent form.

When the sick-listed worker is allocated to the intervention group, the insurance physician of the intervention team will be asked to investigate any (medical) contra-indications for participation in the participatory supportive RTW program, e.g. severe co-morbidity because of a terminal disease, a severe psychiatric disorder, or a serious cardio-vascular disease and/or the absence of work abilities due to medical reasons for at least 3 months. In case of an identified contra-indication, the study participant will not be referred to the participatory supportive RTW program. However, according to the intention-to-treat-principle, the participant will remain in the intervention group.

### Recruitment of participants

Workers without a permanent employment contract who have applied for a sickness benefit at the Dutch SSA and are sick-listed between one and two weeks, will receive an invitation package from the medical advisor of the SSA, on behalf of the researchers. It contains an invitational letter, a flyer with more details about the study, a consent form and a short questionnaire with a return envelope. A weekly query of the SSA database will be used for the recruitment of eligible workers.

The short screening questionnaire consists of six questions. The sick-listed worker will be asked to fill in whether he or she is interested to participate in the study and to indicate the day he or she applied for a sickness benefit. The Distress Screener, developed by van Oostrom and colleagues [30], will be used as a quick scan for early identification of distress, i.e. three questions of the Four-Dimensional Symptom Questionnaire (4DSQ) will be used to assess the degree of perceived mental health problems. Finally, the sick-listed worker will be asked whether he or she has ‘certainly not/probably not/maybe/probably/certainly’ the intention to RTW if health complaints are still experienced.

In case the sick-listed worker wants to participate and meets the eligibility criteria, he or she will be contacted by the researcher or research assistant for a first intake by telephone. During this intake more information about the study will be given. When the sick-listed worker has indicated to ‘maybe/probably/certainly’ have the intention to RTW despite health complaints, the sick-listed worker will be invited to participate in the RCT. Using the described exclusion criteria, the researcher or research assistant will decide whether the sick-listed worker is able to participate.

In case the sick-listed worker is able to participate, an intake appointment will be planned at the nearest participating front office of the SSA. During the intake, randomisation will be performed after signing informed consent and fulfilling the baseline questionnaire by the participant.

**Figure 1:**
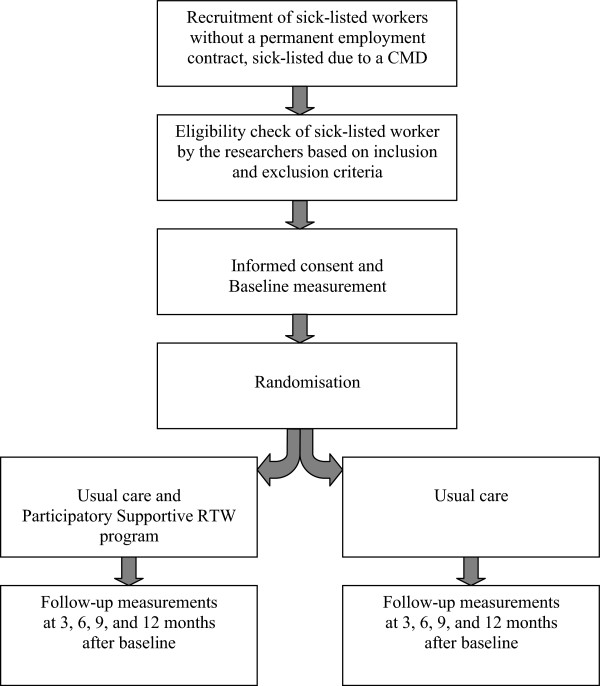
Design of the randomised controlled trial.

In Figure 1 the consecutive steps in the study design are summarized.

### Usual occupational health care

After the sickness benefit application by the sick-listed worker, a RTW coordinator of the SSA will note down the reason for reporting sick and investigates why the sick-listed worker thinks he or she is not able to perform his or her job anymore. An insurance physician of the SSA will decide whether to approve the sickness benefit claim on the basis of a medical assessment. During this assessment, the insurance physician will make a (medical) problem analysis with an advice about recovery, i.e. health promotion and RTW possibilities [8].

In case the sickness benefit claim is approved, the insurance physician, the RTW coordinator and a labour expert of the SSA together are responsible for RTW coaching for the duration of the sickness benefit. The sick-listed worker will be guided according to the Dutch guidelines for OHC. He or she is obligated to visit the OHC professionals and to cooperate with regard to recovery and RTW. The sickness benefit will end when the worker is no longer work disabled [21].

### The participatory supportive RTW program

The aim of the participatory supportive RTW program is to make a consensus-based action plan to achieve RTW. There are four main stakeholders. These stakeholders are the participant, i.e. the sick-listed worker himself/herself, the insurance physician of the SSA, a RTW coordinator of the SSA who guides the vocational rehabilitation process and a labour expert of the SSA who coaches the participant and the RTW coordinator in the development of a RTW action plan.

The labour expert is responsible for equal involvement of both the participant and the RTW coordinator of the SSA in making a RTW action plan with the aim to achieve consensus. Similar process guidance by a trained coach was earlier successfully applied in a participatory RTW program for sick-listed unemployed workers and temporary agency workers with musculoskeletal disorders [31].

**Figure 2:**
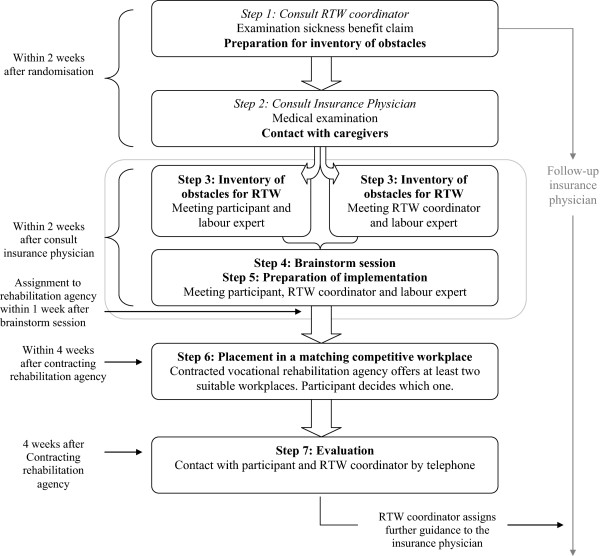
Content of the participatory supportive RTW program.

Figure 2 gives a schematic overview of the content of the participatory supportive RTW program.

#### Guidance by the RTW coordinator and insurance physician

Within two weeks after the intake appointment at the SSA, the participatory supportive RTW program will start with an examination of the sickness benefit claim by the RTW coordinator and a medical assessment by the insurance physician conform usual OHC. In addition, the participant will receive a take-home assignment from the RTW coordinator. He or she will be asked to list obstacles for RTW as a preparation for the first meeting with the labour expert. Obstacles can be both work related or non-work related.

A strong cooperation and communication between the insurance physician, the GP and mental health care specialists are required. Therefore, the insurance physician will contact the caregivers of the participant right after the first medical assessment by telephone to make sure that the participant is given no conflicting advice and to agree upon treatment and RTW options.

#### Inventory of obstacles for RTW

The goal of the meeting between the participant and the labour expert is to identify obstacles for RTW, from the perspective of the participant. The inventory of obstacles for RTW, filled in by the participant as a take-home assignment, will be used as a starting point. During the identification of obstacles, all aspects of disability should be taken into account, i.e. equal attention should be paid to (perceived) biological, psychological and social obstacles [32]. At the end of this meeting the identified obstacles will be prioritized on the basis of frequency and (perceived) severity of the obstacle. In a separate meeting between the labour expert and the RTW coordinator, obstacles for RTW for the participant from the perspective of the RTW coordinator will be identified and prioritised.

#### Brainstorm session

At the start of the brainstorm session, the labour expert will summarize the three main obstacles identified by the participant and by the RTW coordinator, resulting in maximally six prioritised obstacles. According to the nominal group technique [18], both the participant and the RTW coordinator will then be asked to think of as many as possible work-related or non-work-related solutions to overcome each prioritised obstacle for RTW. The proposed solutions will be judged on the basis of feasibility to solve the barrier. It is important to determine who is responsible for the fulfilment of each solution, and when this should be organized and finalized. Subsequently, the participant and the RTW coordinator are asked to think of suitable work, i.e. type of work, content and duration of tasks, time path and necessary preconditions. The ultimate goal of this session is to achieve consensus between the participant and the RTW coordinator about solutions to overcome obstacles for RTW and about suitable work.

The inventory of obstacles and the brainstorm session are based on an existing participatory RTW program [18,19,33].

#### Preparation of implementation

The labour expert will underline the participant’s own responsibility to search for suitable work. The formulation of suitable work solutions can help the participant to explore the labour market.

Within two days after the brainstorm session, the labour expert will make a written report of the prioritised obstacles and the consensus-based solutions for RTW, including a concrete work profile in which the content of suitable work tasks, a time path and necessary preconditions are summarised. This action plan for RTW will be presented to the insurance physician who will consider if the proposed suitable work solutions are in line with the physical and mental work capacities of the participant. After comments of the insurance physician have been integrated in the report, it will be sent by the labour expert to the participant, the insurance physician and the RTW coordinator. If necessary, the insurance physician will communicate this action plan for RTW to other caregivers of the participant to promote collaboration.

#### Placement in a matching competitive workplace

The participant will be supported in the search for a suitable workplace by one of the three rehabilitation agencies that participate in the study. Intervention group participants will be equally assigned to the participating agencies.

After receiving the written action plan for RTW, the RTW coordinator will contact the case manager of the assigned rehabilitation agency and will inform the case manager about necessary preconditions for RTW. The rehabilitation agency will receive a copy of the action plan for RTW.

Within four weeks, the agency has to offer at least two suitable workplaces, with a contract period of at least three months, matching with the formulated consensus-based action plan for RTW and taking into account the participant’s preferences. The employment contract has to result in at least 50 percent of the earnings of the participant’s last job. Alternatively, placement for a maximum of three months with ongoing sickness benefit is possible, but only when after these three months the employment contract meets the requirements mentioned above. In that case, there should be an intention to offer the participant a (temporary) employment contract. A financial reward will be given by the SSA to the rehabilitation agency for the job hunting and/or for the actual placement in a matching workplace. The participant will be actively involved in the job searching.

The case manager of the rehabilitation agency is responsible for proper guidance of the participant. If required, the case manager will visit the workplace to instruct and advise the participant. And, if necessary, the supervisor and/or colleagues at the workplace can be informed by the case manager about how to guide the participant at the workplace.

#### Evaluation

Four weeks after the start of the job search by the rehabilitation agency, the RTW coordinator will contact the participant and the case manager of the agency by telephone to inform whether placement in a workplace has been successful. The RTW action plan will be evaluated and, if necessary, the action plan will be adapted to new circumstances. The RTW coordinator summarizes findings in a final report.

In case the assigned rehabilitation agency has not been able to offer a suitable workplace, the other two rehabilitation agencies participating in the project will also get the opportunity to search for suitable vacancies.

Six weeks and three months after placement in a workplace, the case manager of the rehabilitation agency will evaluate the program with the participant, and will send a report with a summary of the most important findings to the RTW coordinator of the SSA.

#### Training of the professionals

Instruction will be given to all intervention teams by the researchers. At each participating SSA office instruction takes place by means of a presentation and role plays during one session of approximately three hours. In the beginning of this session all professionals will receive a syllabus with detailed information about the program, the protocol, practical summaries and schemes and practice material. A few months after the first participants have enrolled in the intervention, the researchers will visit every participating intervention team for a follow-up session to evaluate the first cases and to discuss difficulties in applying the protocol in daily practice.

### Use of co-interventions

Co-interventions cannot be avoided. It is possible that the study participants will receive other interventions. In both the intervention and control group received co-interventions will be monitored in each follow-up measurement.

### Outcomes

#### Effect evaluation

The primary outcome measure is the duration until first sustainable RTW in competitive employment. This is defined as the duration in calendar days from the day of enrolment in the study until first sustainable RTW in a competitive job for at least 28 consecutive calendar days without partial or full recurrence of sickness absence. In line with Crowther et al. [25], competitive employment is defined as a full or part-time position held by the worker in a regular work setting, for which payment is received at the market rate.

According to the Dutch Sickness Benefit Act, recurrence of an accepted sickness benefit claim within 28 calendar days after ending of the previous benefit is considered as belonging to the preceding sickness benefit period, on condition that it is due to the same (or related) disorder. Although for sick-listed workers without a permanent employment contract ending of the sickness benefit not automatically results in RTW, it was chosen to mark RTW as sustainable only when the participant returned to work for at least these 28 calendar days.

RTW data, i.e. work resumption in regular (paid) work, are registered continuously by the Dutch SSA and will be collected from the SSA database after twelve months follow-up.

In addition, with a self-administered questionnaire the participant will be asked whether he or she has worked in (un)paid labour in the last three months. If the participant did RTW, he or she will be asked to specify the period in which RTW has taken place and the average working hours per week.

Secondary outcome measures are:

– *RTW in any type of work*

 In addition to the primary outcome measure, the duration until first RTW in any type of work will be measured, i.e. paid work, unpaid work and work with ongoing supportive benefit.

– *Duration of the sickness benefit period*

 For workers without a permanent employment contract, it is possible that the sickness benefit ends, before full RTW is achieved. The worker can be recovered from illness or functional limitations (assessed with regard to last or previous work) without actual RTW, because the worker has no workplace to return to. Therefore, in line with Vermeulen and colleagues, the duration of the sickness benefit period will be assessed as well. This is defined as the duration of the sickness benefit from the day of enrolment until ending of the sickness benefit for at least 28 consecutive calendar days [21]. Additionally, the total number of days of sickness benefit during follow-up will be calculated. Awarded sickness benefit claims during follow-up are only included in the calculation when the participant is sick-listed due to the same (or related) mental disorder [21]. Data on sickness benefit will be collected from the SSA database and by self-report of the participants.

– *Work status*

 Work status is defined as the average number of hours worked per week during the one-year follow-up. In addition to a self-administered questionnaire, the SSA database will be used to collect this information.

– *Severity of mental disorder symptoms*

 Severity of mental disorder symptoms will be assessed using the Four-Dimensional Symptom Questionnaire (4DSQ) [34].

– *Perceived general health status*

 Using the Dutch translation of the SF-36 [35] perceived general health status will be measured.

– *Quality of life*

 Quality of life will be measured using the Dutch translation of the Euroquol questionnaire [36].

– *Attitude, Social influence, and self-Efficacy (ASE)*

 For the development of earlier participatory RTW programs the Attitude-Social influence-self-Efficacy (ASE) model was used as an underlying theoretical framework [37,38]. In these studies the ASE constructs were assessed using a questionnaire developed by Van Oostrom and colleagues [39]. In this study we will make use of the same questionnaire.

– *Work limitations*

 Work limitations will be measured with the Dutch translation of the Work Limitations Questionnaire (WLQ) [40].

### Prognostic measures

Demographic characteristics, information regarding last work, type of worker before reporting sick and reason for reporting sick will be assessed with a self-administered questionnaire at baseline.

At the same time, the way health complaints influence vocational rehabilitation will be assessed. This will be measured with questions belonging to the subscale ‘Fear-avoidance beliefs’ of the Dutch Work Reintegration Questionnaire (WRQ) [41,42].

During follow-up, in case full RTW is not (yet) achieved, RTW expectations are measured. With a self-administered questionnaire, participants will be asked to indicate the period within they think it is possible to achieve full RTW (in ‘own’ work or other).

In addition, in each questionnaire participants will be asked whether they received RTW coaching by the SSA and whether they were treated for their health complaints. In case the participant indicates that he or she received RTW coaching by the SSA, questions will be asked about efforts made by the SSA to reintegrate the participant, e.g. investments in education or training and contracting a rehabilitation agency. The participant will be asked to rate the efforts of the SSA on a scale of one to ten. Also, when applicable, the participant will be asked to describe the treatment for his/her health complaints.

### Economic evaluation

Direct and indirect costs will be measured to conduct an economic analysis from the social insurer’s perspective and the societal perspective.

Costs for health care utilization, OHC and investments in vocational rehabilitation support made by the SSA are considered as direct costs. Examples of investments made by the SSA are training or education, interventions aimed at health promotion and contracting a rehabilitation agency to search for a workplace.

Indirect costs are related to paid sickness benefits. In case an employee becomes sick-listed, loss of productivity is normally considered to be part of the indirect costs. However, because sick-listed temporary agency workers, unemployed workers and workers with an expired fixed-term employment contract no longer have an employment contract, loss of productivity does not result in indirect costs [23]. Unemployed workers and workers whose employment contract ended during sickness absence have no workplace (anymore), which means there is no loss of productivity. The sick-listed temporary agency worker will, in case of sick-listing, be replaced with a healthy worker, which results in no productivity loss for the company concerned.

Data on paid sickness benefits and costs for investments made by the SSA will be collected from the SSA database and the worker’s files after one year follow-up. Data on OHC by the SSA professionals, i.e. number of consults during follow-up and type of OHC professional, will be collected from the SSA database and the medical files. Health care utilization will be measured by the Trimbos/iMTA questionnaire for Costs associated with Psychiatric Illness (Tic-P) [43]. The Tic-P is developed to measure health care utilization of people with mental illnesses. It quantifies the number of visits to different health care providers. Prices for different health care services suggested in guidelines for economic evaluation in the Netherlands will be used to value the health care consumption [44].

### Process evaluation

Based on the framework of Steckler and Linnan a process evaluation will be conducted [45]. The aim of the process evaluation is to determine the compliance with the intervention protocol, the feasibility of the participatory supportive RTW program and to assess satisfaction with the OHC guidance in accordance to this program. Three months after the participant has been assigned to the intervention group, the participant, the OHC professionals of the intervention team and the case manager of the rehabilitation agency will all receive a questionnaire. The OHC professionals and the case manager of the rehabilitation agency will be asked whether the intervention was applied according to the protocol. Additionally, they will be asked about applicability, compliance, satisfaction and barriers regarding implementation of the participatory supportive RTW program. Also the participants will be asked about their satisfaction with the participatory supportive RTW program. These questions are based on the Patients Satisfaction with Occupational Health Services Questionnaire (PSOHSQ) [46] and will be included in the three months questionnaire.

During the participatory supportive RTW program, standardized schemes will be used by the OHC professionals to describe identified barriers for RTW, the formulated solutions, the resulting consensus-based action plan for RTW and a final report. These schemes will be used to collect additional data about the implementation of the participatory supportive RTW program.

An overview of the measures and measurement instruments, including a time path for all measurements, is presented in Table 1.

**Table 1:** Overview of measurements and time path

**Measurement**			** Time path**		
	**Baseline (T0)**	**3 months (T1)**	**6 months (T2)**	**9 months (T3)**	**12 months (T4)**
*Prognostic measures:*					
Demographic characteristics (e.g. age, gender)	X				
Last work (shifts, hours)	X				
Type of worker before reporting sick	X				
Reason reporting sick	X	X			
Interference of complaints (WRQ)	X				
RTW expectations	X	X	X	X	X
RTW interventions	X	X	X	X	X
Satisfaction with OHC	X	X	X	X	X
Health care interventions	X	X	X	X	X
*Primary outcome measure:*					
Duration until first sustainable RTW	X	X	X	X	X
*Secondary outcome measures:*					
Duration of sickness benefit	X	X	X	X	X
Work status	X	X	X	X	X
Severity of mental disorder symptoms (4SDQ)	X		X		X
Perceived general health status (SF-36)	X		X		X
Quality of life (Euroqol)	X		X		X
ASE determinants (ASE questionnaire)	X		X		X
Work limitations (WLQ)			X		X
Health care utilization (Tic-P)	X	X	X	X	X
Patient satisfaction* (PSOHSQ)		X			

*Patient satisfaction with occupational health care services is only measured in the intervention group (as part of the process evaluation).

### Data collection

The baseline questionnaire will be filled in during the intake appointment at the SSA, after signing informed consent. All other questionnaires will be filled in online, unless the participant prefers to receive a hard copy by postal mail.

Participants will receive questionnaires at baseline and after three, six, nine and twelve months.

In case questionnaires will not be returned within two weeks after the questionnaire is sent, the researcher will contact the participant by telephone to inform whether the participant has been able to complete the questionnaire and to ask the participant, if possible, to complete the questionnaire timely. In case the participant returns the questionnaire, but the received questionnaire is incomplete, the researcher will also contact the participant by telephone. The remaining questions will be repeated by the researcher, so that the questionnaire can be completed by the participant.

In addition to the questionnaires, after one year follow-up data regarding RTW, sickness absence, diagnosis, OHC interventions and investments made by the SSA will be obtained from the SSA database and the medical file of the worker at the SSA. These data will be checked with the self-reported information in the questionnaires.

### Sample size

Time to first sustainable RTW in a competitive job is the primary outcome measure for the power calculation. Based on a recent study on a participatory RTW intervention for temporary agency workers and unemployed workers with musculoskeletal disorders [22] a Hazard Ratio (HR) of 2.0 is assumed to be the minimal clinical and societal relevant ratio. This indicates that the participants in the intervention group RTW twice as quickly compared to the participants in the control group. Furthermore, it is assumed that a minimum of 2/3 of the participants will achieve first sustainable RTW during the first twelve months of the follow-up period [22]. Based on a power of (1-β=) 0.80 and a two-sided significance level of 0.05 (α) a sample size of 100 participants (n = 2 x 50) is needed. Next, potential clustering of cases guided by the same team of OHC professionals is taken into account. To correct for potential clustering of cases an ICC of 0.05 is used and the minimal number of teams is assessed: eight teams of OHC professionals who are trained in guidance according to the participatory supportive RTW program and eight teams of OHC professionals who deliver only usual OHC. Furthermore, based on comparable research [47], a loss to follow-up of 20% is expected. This results in a requisite number of 172 participants (n = 2 x 86).

### Randomisation procedure

Randomisation will take place on participant level. In line with previous research by Vermeulen et al. [21] pre-stratification of participants is based on information about type of worker before reporting sick, i.e. unemployed worker, temporary agency worker or fixed-term contract worker. To ensure an equal distribution of control group participants and intervention group participants in the three different SSA districts, participants will also be pre-stratified on district-level. Schemes with random permuted numbers will be used by the principal investigator to generate separate block randomisation tables with fixed block sizes of four.

Randomisation takes place during the intake appointment at the SSA office. After the informed consent form is signed and the baseline questionnaire is completed by the participant, the assistant of the SSA contacts the research assistant at the VU Medical Centre to perform the randomisation. The research assistant of the VU Medical Centre uses the block randomisation table of the correct stratum to determine the randomisation result. The participant will be informed immediately about the randomisation result, intervention or control group, and the consecutive steps. Participants who are allocated to the intervention group will be assigned to an intervention team of the corresponding SSA office for guidance according to the participatory supportive RTW program in combination with usual OHC. These intervention teams will not be involved in the guidance of control group participants. Control group participants will be assigned to a team of the corresponding SSA office that is not familiar with the intervention program.

### Blinding

The OHC professionals who perform the intervention cannot be blinded for the allocation of participants to the intervention group, because they will need to know when to apply the intervention. Also the OHC professionals who are not trained in the participatory supportive RTW program will be informed when a participant is allocated to their team for usual OHC. Randomisation of participants will take place on participant level and participants of both the control group and the intervention group will receive OHC by OHC professionals working at the same office. Therefore, blinding the professionals for the randomisation result is impossible.

Because participants need to be informed at least briefly about the content of usual OHC and the participatory supportive RTW program before they are able to sign an informed consent, they can as well not be blinded for the randomisation result. Also blinding the participants for the outcome measures will be impossible, as most of the outcomes are self-reported. Bias caused by a lack of blinding will however be limited for the measurement of the duration until first sustainable RTW, the primary outcome measure of this study, and the duration of the sickness absence period, as in addition to the questionnaires the SSA database will be used to measure these outcomes.

To guarantee blinded analyses of the collected data by the researcher, the data will be entered into a database by a research assistant using a unique research number for each participant.

### Contamination

Since the intervention teams will not be involved in the guidance of control group participants, contamination will be limited. However, since trained and non-trained OHC teams are working at the same department, non-trained professionals could still be influenced in their usual practice by the intervention teams. Contamination of usual care and the participatory supportive RTW program may also appear when participants have already received usual OHC before they are assigned to the intervention group. Therefore, sick-listed workers who have already received usual OHC cannot participate in the study.

### Statistical analysis

After randomisation, participants will remain in the group (intervention group or control group) they are allocated to, according to the intention-to-treat-principle. Descriptive statistics will be used to check for dissimilarities of prognostic factors in the two groups at baseline. If necessary, analysis will be adjusted. A comparison of intention-to-treat-analysis to per-protocol analysis will be used to determine whether protocol deviations might have caused bias. All statistical analysis will be performed at participant level.

#### Effect evaluation

The duration until first sustainable RTW in a competitive job in both groups will be described by using the Kaplan-Meier method. The Cox proportional hazard model will be used to estimate differences in RTW between the intervention group and the control group, expressed in hazard ratios (HR) for sustainable RTW and the corresponding 95% confidence intervals. Differences between both groups in total number of days at work and total days of sickness benefit during follow-up will be analysed with a general linear model. Differences in other secondary outcome measures will be analysed with the use of longitudinal random coefficient analysis. Clustering of participants within the OHC teams will be taken into account.

#### Economic evaluation

Cost-effectiveness will be assessed from both the social insurer’s perspective and the societal perspective by dividing the incremental costs by the incremental effects. The incremental cost-effectiveness ratio (ICER) represents the additional costs needed to gain one extra unit of effect in the intervention group compared to the control group. Cost-utility will be measured by dividing the differences in total costs by the difference in QALYs between the two groups.

A cost-benefit analysis will be conducted from the societal perspective. The net monetary benefit will be calculated by subtracting the difference in total costs between the two groups from the differences in productivity gain. Return on investment will be measured by dividing the incremental benefit (gain minus costs) by the incremental costs of the investment.

Bootstrapping will be used to estimate uncertainty surrounding the incremental costs. Confidence intervals (95%) around the mean costs differences will be computed by bias corrected and accelerated bootstrapping.

## Discussion

The participatory supportive RTW program combines elements of a participatory RTW program, integrated care and direct placement in a competitive job in order to improve the RTW of workers without a permanent employment contract who are sick-listed due to a CMD.

The cost-effectiveness of the participatory supportive RTW program will be examined in a RCT. This paper describes the study design.

### Strengths of the study

An important strength of the study is that it pays attention to sick-listed workers without a permanent employment contract who experience more barriers for RTW compared to sick-listed employees. Moreover, the participatory supportive RTW program was specifically tailored to an important diagnose group, namely the CMDs.

A second strength of the study is that it is a pragmatic RCT, as the intervention is performed in daily practice. Another strength is that the study includes a process evaluation to determine the feasibility of the participatory supportive RTW program within the Dutch SSA system and satisfaction with this program. Because the RCT is conducted in daily practice and a process evaluation is included, the study will provide important information for possible future implementation of the RTW program.

Finally, the collection of data on RTW and duration of sickness benefit via the SSA database can be seen as an important strength of the study. This minimizes possible bias that can be caused by self-report of the participants and the OHC professionals.

### Limitations of the study

A first limitation of the study is that generalizing the results of the cost-effectiveness of this program to other countries can be difficult, especially in countries where sick-listing is not possible without an employment contract. The participatory supportive RTW program is specifically tailored to the Dutch context. In the Netherlands the SSA is responsible for sickness absence counselling of sick-listed workers who have no (longer an) employment contract.

Secondly, because of pragmatic reasons the follow-up period of participants is one year after enrolment in the study. To measure (long-term) cost-effectiveness of the intervention, a longer follow-up period would have been more preferable.

A third limitation is the absence of a pilot study prior to the RCT. A pilot study could have provided important information on how the programs activities of the participatory supportive RTW program fit in the daily activities of the OHC professionals at the Dutch SSA. In addition, a pilot study would have provided more information about the feasibility of placement in a suitable and competitive workplace by the participating rehabilitation agencies. As an alternative, interviews were held with different representatives of the SSA to gather information about the daily practice within their department and about the different occupational roles within the teams of OHC professionals. In addition, during the training in the participatory supportive RTW program, the professionals were asked if there were any flaws in the intervention program that could harm a successful implementation. Small adaptations were made to improve the practicability of the program. Also the representatives of the rehabilitation agencies were asked to judge the feasibility of their role in the participatory supportive RTW program on the basis of their experience with job hunting in de Dutch labour market.

The OHC professionals and the participants will not be blinded for the group allocation in the RCT, which can be seen as another limitation of the study. Because of the allocation of participants of both groups to separate teams of OHC professionals working at the same SSA office, blinding of these professionals will not be possible. Prior to the randomisation, participants will be informed about the nature of usual OHC and the participatory supportive RTW program, so that they can give an informed consent. Therefore, blinding of the participants for the randomisation result will not be possible either.

Finally, the study population is limited to sick-listed workers who have at baseline the intention to RTW despite their health complaints. Earlier research on the effectiveness of a participatory RTW approach already indicated that sick-listed workers who believe they should be fully recovered before they RTW require another intervention approach [20,22]. Little is known about successful RTW interventions for sick-listed workers who do not intend to RTW if they still face health complaints. For that reason, we will conduct a separate cohort study to identify prognostic factors for the duration until RTW for this particular group.

### Impact of study findings

In order to overcome an important obstacle for the RTW of most sick-listed workers without a permanent employment contract, which is the absence of a workplace to return to, placement in a competitive job was incorporated into the RTW program. In the field of OHC research direct placement in a competitive job has been extensively evaluated as part of IPS programs for the severely mentally ill. IPS has been robustly validated by research in the United States [24,25,48] and receives growing attention in Europe [49]. This study will increase knowledge about the effectiveness of this approach for workers who are sick-listed due to less severe and more common mental disorders.

Moreover, the results of this RCT on the cost-effectiveness of the participatory supportive RTW program will demonstrate whether this program is effective in improving RTW for a vulnerable group of sick-listed workers and whether it will outweigh the societal costs and the expenditures made by the Dutch SSA. Current figures of the Dutch SSA show that sick-listed workers without a permanent employment contract run a greater risk of a long term disability claim compared to sick-listed employees [7], resulting in high costs related to disability benefit payment. Mental disorders are the most frequently diagnosed disorders within this group [9]. Henderson states in his editorial on long term sickness absence that this longer absence is associated with a reduced probability of eventual RTW and relates this to subsequent social and economic deprivation [50]. If the participatory supportive RTW program proves to be cost-effective, the social security system, the sick-listed worker and society as a whole will benefit. For social security and society, a cost-effective RTW program will lead to a reduction of costs related to long term sickness absence. For the sick-listed worker a cost-effective RTW program results in earlier sustainable RTW, which can be associated with both social and health benefits [26].

Results of this study will become available in 2015.

## Competing interests

The authors declare they have no competing interests.

## Authors’ contributions

LL drafted the manuscript. LL, SJV and FGS developed the study design and intervention protocols. LL, SJV and FGS are responsible for the general coordination of the study. LL is responsible for the data collection. All others have read and corrected draft versions of the manuscript and approved the final manuscript.

## Pre-publication history

The pre-publication history for this paper can be accessed here:

http://www.biomedcentral.com/1471-2458/14/594/prepub
